# Significance of Mast Cell Formed Extracellular Traps in Microbial Defense

**DOI:** 10.1007/s12016-021-08861-6

**Published:** 2021-05-22

**Authors:** Daniel Elieh Ali Komi, Wolfgang M. Kuebler

**Affiliations:** 1 Cellular and Molecular Research Center, Cellular and Molecular Medicine Institute, Urmia University of Medical Sciences, Urmia, Iran; 2 Institute of Physiology, Charité - Universitätsmedizin Berlin, Charité Campus Mitte (CCM), Berlin, Germany; 3 German Center for Cardiovascular Research (DZHK), Partner site Berlin, Berlin, Germany; 4 Departments of Surgery and Physiology, University of Toronto, Toronto, Canada

**Keywords:** Extracellular traps, LL-37, Mast cells, Microbial defense, ROS, Tryptase

## Abstract

Mast cells (MCs) are critically involved in microbial defense by releasing antimicrobial peptides (such as cathelicidin LL-37 and defensins) and phagocytosis of microbes. In past years, it has become evident that in addition MCs may eliminate invading pathogens by ejection of web-like structures of DNA strands embedded with proteins known together as extracellular traps (ETs). Upon stimulation of resting MCs with various microorganisms, their products (including superantigens and toxins), or synthetic chemicals, MCs become activated and enter into a multistage process that includes disintegration of the nuclear membrane, release of chromatin into the cytoplasm, adhesion of cytoplasmic granules on the emerging DNA web, and ejection of the complex into the extracellular space. This so-called ETosis is often associated with cell death of the producing MC, and the type of stimulus potentially determines the ratio of surviving vs. killed MCs. Comparison of different microorganisms with specific elimination characteristics such as *S pyogenes* (eliminated by MCs only through extracellular mechanisms), *S aureus* (removed by phagocytosis), fungi, and parasites has revealed important aspects of MC extracellular trap (MCET) biology. Molecular studies identified that the formation of MCET depends on NADPH oxidase-generated reactive oxygen species (ROS). In this review, we summarize the present state-of-the-art on the biological relevance of MCETosis, and its underlying molecular and cellular mechanisms. We also provide an overview over the techniques used to study the structure and function of MCETs, including electron microscopy and fluorescence microscopy using specific monoclonal antibodies (mAbs) to detect MCET-associated proteins such as tryptase and histones, and cell-impermeant DNA dyes for labeling of extracellular DNA. Comparing the type and biofunction of further MCET decorating proteins with ETs produced by other immune cells may help provide a better insight into MCET biology in the pathogenesis of autoimmune and inflammatory disorders as well as microbial defense.

## Introduction

**Fig. 1:**
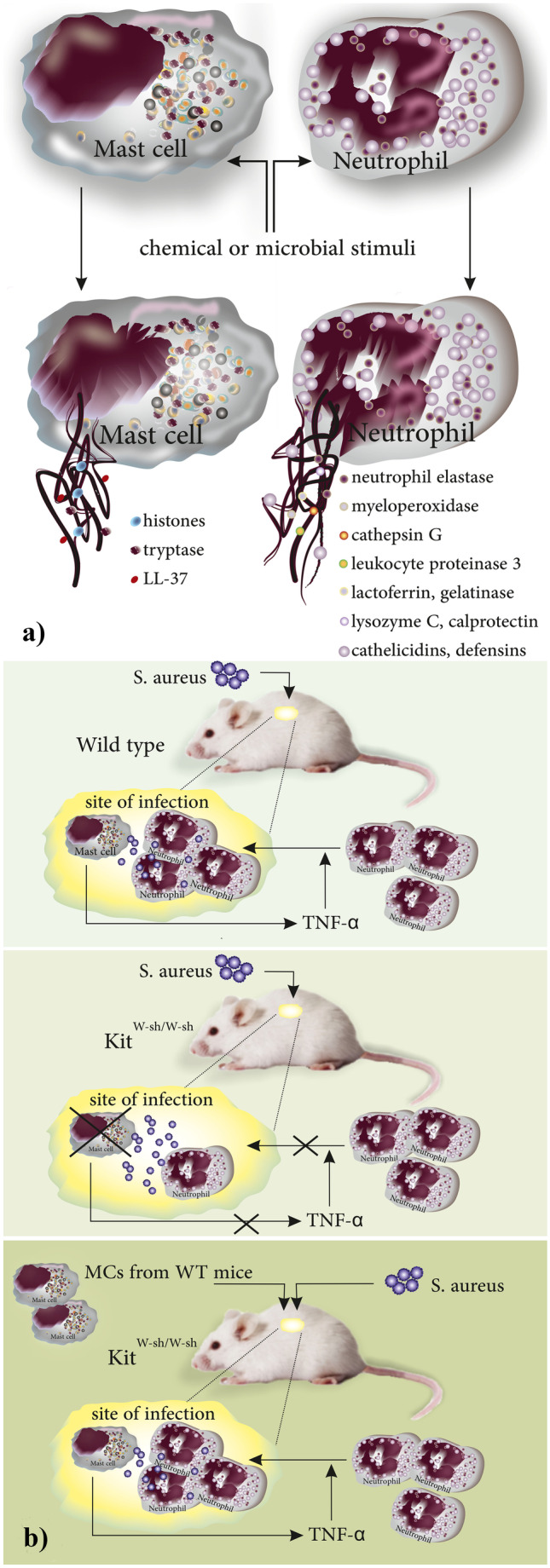
**a** structure of ETs, ET-associated proteins, and the nature of DNA depend on the producing cell types. **b** Role of MCs in antimicrobial defense against *S. aureus*: MCs release TNF-α which is a critical neutrophil attractant to the site of infection. MCs from MC-deficient *Kit*^W−sh/W−sh^ mice cannot effectively attract neutrophils when compared to the wild type Kit^+/+^ MCs. When WT MCs are injected to MC-deficient *Kit*^W−sh/W−sh^ mice, they restore their ability to eliminate the bacteria by recruiting neutrophils to the site of infection

**Fig. 2:**
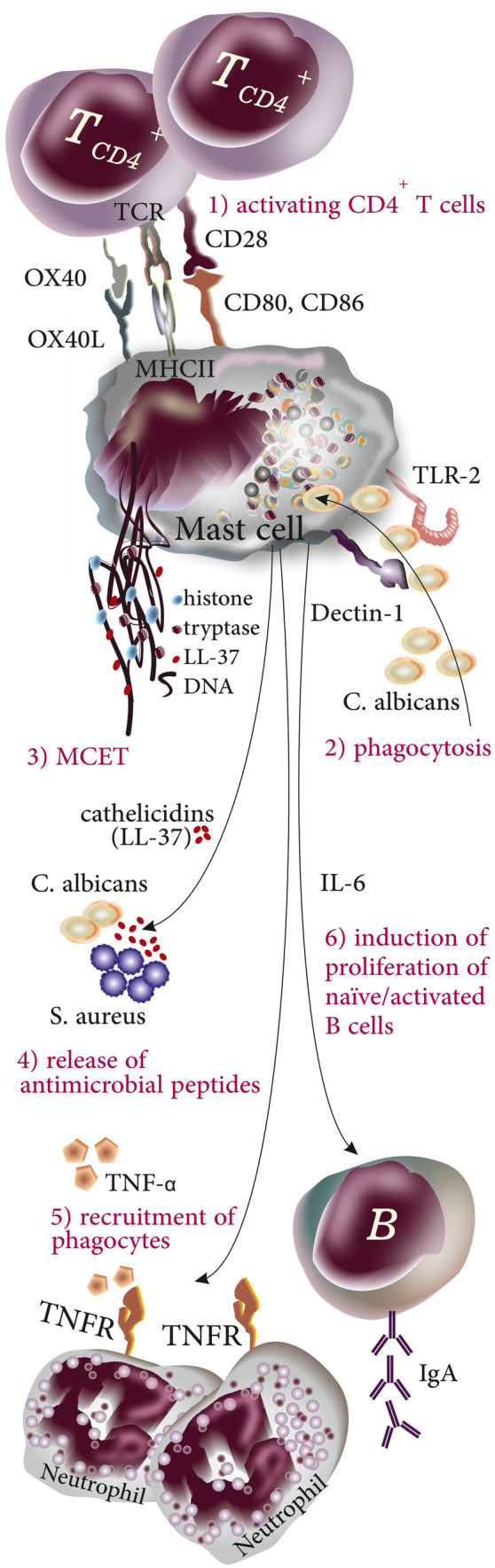
Intracellular and extracellular mechanisms of microbial defense used by mast cells. (1) MCs act as antigen presenting cells by expressing MHC class II molecules and costimulatory molecules to activate CD4^+^ T cells and support the orchestration of adaptive immune responses; (2) MCs can act as phagocytes by directly engulfing invading pathogens and killing them in phagolysosomes; (3) MCs produce MCETs consisting of DNA, histones, LL-37, and tryptase to trap and immobilize invading pathogens; (4) MCs produce and release antimicrobial peptides such as the cathelicidin LL-37; (5) MCs effectively recruit other phagocytes to the site of infection by releasing cytokines such as TNF-α for neutrophil recruitment; and (6) MCs play a role in induction of proliferation in B cells by releasing cytokines and surface receptors

Formation of extracellular traps (ETs) by several types of leukocytes occurs as a late antimicrobial response to the presence of microbial invaders (in vivo) or special chemicals (mostly reported in in vitro experiences) [1, 2]. Although ET formation was primarily described as a mechanism used by leukocytes in microbial defense, ETs were later shown to be associated with several non-infectious pathologies including psoriasis, systemic lupus erythematosus (SLE), liver damage, acute pancreatitis, and cancer metastasis [2–6]. ETs, the thread-like complexes of decondensed DNA (nuclear or mitochondrial DNA [7]) with attached proteins from cytoplasmic granules, were first reported in neutrophils to act as an extracellular mechanism in microbial defense [8]. The formation of ETs in leukocytes results in the cell death of the leukocyte which from a molecular point of view is neither necrosis nor apoptosis [9]. Extracellular traps gained attention when they were reported to be produced by other myeloid cells such as monocytes [10] or eosinophils [11]. The molecular structure of ETs depends on the type of the producing cell and the stimuli; for instance, neutrophil ETs (NETs) are comprised of neutrophil elastase (NE), myeloperoxidase (MPO), cathepsin G, leukocyte proteinase 3 (PR3), lactoferrin, gelatinase, lysozyme C, calprotectin, cathelicidins, and defensins [9]. In contrast, mast cells (MCs), another innate immune cells, produce ETs (MCETs) containing histones, tryptase, and LL-37 [12] (Fig. 1a). The main biologic functions of these biomolecules and mediators are listed in Table 1. MCs are granulated leukocytes of innate immunity that differentiate in target tissues from CD117 + /CD34 + progenitors released from the bone marrow [13, 14]. Under the influence of growth factors such as stem cell factor (SCF), IL-3, IL-4, IL-9, IL-10, IL-33, and TGF-β [15], MC progenitors differentiate in functional mature cells that respond to a variety of environmental stimuli owing to expression of receptors including toll-like receptors and receptors to Fc portion of antibodies (such as FcεRI:IgE or FcγR: IgG) [16–18]. Beyond their classic role in allergic and anaphylactic reactions [19], MCs play an important role in microbial defense [12]. At very early steps of microbial invasion, MCs effectively recruit neutrophils to the site of infection by releasing TNF-α which is a preformed and stored mediator of MCs [20]. The results of experimental infection with *S. aureus* in MC-deficient *Kit*^W−sh/W−sh^ mice and corresponding wild type (WT) littermates or reconstitution of MC-deficient mice with MCs derived from WT mice showed that (a) In *Kit*^W−sh/W−sh^ mice recruitment of neutrophils and elimination of bacteria were impaired, (b) reconstituting the MC population in *Kit*^W−sh/W−sh^ mice by injection of MCs from WT mice could restore their ability to eliminate the bacteria, and (c) exogenous TNF-α could compensate the partial ineffectiveness of MC-deficient mice in recruiting neutrophils to the cite of infection supporting the notion that MC-released TNF-α participates actively in microbial defense [21] (Fig. 1b). MCs utilize both intracellular (including phagocytosis) and extracellular mechanisms (mainly via release of peptides with antimicrobial properties) for the elimination of invading pathogens [12, 22]. Additionally, MCs activate CD4^+^ T cells by acting as antigen presenting cells (APCs). It is now evident that MCs express MHC-II and costimulatory molecules such as OX40L, CD80, and CD86 to activate CD4^+^ T cells (expressing the corresponding receptors including OX-40 and CD28, respectively) and as such, orchestrate adaptive immune responses [23, 24]. Besides, MCs are abundant in B cell localizing areas in lymph nodes and the coculture of these two cell populations revealed that MCs induce the proliferation of both naïve and activated B cells and support their differentiation into IgA producing cells via expressing CD40L and releasing IL-6 [25]. Accordingly, MC-released IL-6 can play a critical role in the activation and proliferation of B cells in vivo [26]. MCs express different types of surface receptors to recognize microbes including TLR-2/Dectin-1 for the detection of *C. albicans* and produce nitric oxide (NO) which possesses cytotoxic effects against microorganisms [27, 28]. The ability of MCs to produce extracellular traps (ETs) was first reported in 2008 [12] (Fig. 2). ETosis of MCs and subsequent cell death can be inhibited by the NADPH oxidase inhibitor diphenyleneiodonium (DPI) indicating a critical role for reactive oxygen species (ROS) in MCET formation [12, 29]. In the following sections, we will review different aspects of MCETs with focus on their structure, microbial and chemical stimuli that induce their formation, their role in restriction of microbial infections, and finally possible involvement in several noninfectious pathologies [30]. Additionally, we will discuss the technical procedure commonly used to stain the different components of MCETs and visualizing them under microscope.

**Table 1:** The main properties and biofunctions of ET-associated proteins in neutrophils and MCs

Producing cell	ET-associated proteins	Main properties and biofunctions of biomolecules and mediators attached to DNA strands	Ref
Neutrophil	Neutrophil elastase (NE)	• A serine protease expressed in primary granules	[95]
		• In humans, NE translocates from azurophilic granules to the nucleus upon formation of NET where it cleaves histones and contributes to chromatin decondensation by partially degrading specific histones	[96]
		• Neutrophils of NE^−/−^ mice produce NETs when stimulated by PMA	[97]
		• Maintains its catalytic ability after being localized to DNA	[98]
		• It is suggested that NE blocking would largely abrogate the protease activity associated with NETs	[99]
	Myeloperoxidase	• Synergies with NE in decondensation of chromatin during NETosis	[96]
		• A granule component of neutrophil that possesses antiviral activity	[100]
	Cathepsin G	• Cleaves the pro-IL-1α precursor and produces more IL-1α through which it activates endothelial cells	[101]
		• Plays a role in platelet activation, platelet aggregation, and dense granule secretion	[102, 103]
	Leukocyte proteinase 3	• Has similar substrates, structural and functional characteristics with NE	[104]
		• it is a neutral protease identified as the principal antigen of antineutrophil cytoplasm autoantibodies (c-ANCA)	[104]
		• Like other NET-associated proteases (NE and cathepsin G), leukocyte proteinase 3 is activated by dipeptidyl peptidase I (DPPI) in mature neutrophils	[105]
	Lactoferrin	• Deprives the bacteria of iron by capturing iron	[106]
		• Polysialic acid modulates the Binding of external lactoferrin in NETs	[106]
		• Binds DNA through interactions of positively charged residues located in the N-terminal with negatively charged DNA	[107]
		• Similar to elastase, lactoferrin is present in the cytoplasm of unstimulated neutrophils but is localized to the cell membrane after 2 h PMA- stimulation	[107]
		• Lactoferrin has been reported to inhibit the release of NET	[106]
	Gelatinase	• Matrix metalloproteinases (MMPs) are zinc-dependent proteases that degrade extracellular matrix and mediate the tissue remodeling	[108]
		• MMP-9 cleaves laminin, chondroitin sulfate, collagen IV, and collagen V	[109]
		• MMP-9 activates the endothelial MMP-2 and drives endothelial dysfunction	[110]
	Lysozyme	• NETs carry lysozyme upon exposure to several microorganisms including *Pseudomonas aeruginosa*	[111]
	Calprotectin	• Structurally is a heterodimer and acts as an effective antifungal component in NETs	[112]
	Cathelicidins	• LL-37 is the only human cathelicidin which is an amphipathic and cationic peptide and has been reported to act as chemotactic AMP. It has immunomodulatory properties	[113]
		• May lose its antimicrobial properties when it binds to DNA	[114]
		• LL-37 induces the formation of NETs in ex vivo experiments	[115]
		• LL-37 has been reported in structure of NETs when neutrophils are exposed to microbes including bacteria and parasites	[116, 117]
	Defensins	• Human β-defensin 1 (hBD-1) is produced by epithelial surfaces and acts mainly against gram-negative bacteria	[118]
		• Mature hBD-1 under influence of thioredoxin is modified and produces redhBD-1 by elimination of disulfide bonds	[119]
		• NET formation induces the production of hBD-2 by keratinocytes in psoriasis	[120]
Mast cell	Histones	• Produced and released as the component of MCETs when MCs are exposed to intra/extracellular pathogens such as *L. monocytogenes, Streptococcus pyogenes,* and *Leishmania*	[12, 59, 63]
		• Histones have been reported to have antimicrobial properties, i.e., H3 and H4 histones cause membrane damage accompanied with blebbing and pore formation, while H2B disrupts the integrity of the cell	[121]
	Tryptase	• The most abundant protease found in the MC secretory granules, that is associated with the pathologies including allergy, inflammation, and tissue remodeling	[122]
		• Tryptase acts as a ligand for protease activated receptor-2 (PAR-2); the cleavage of PAR-2 is the activation mechanism through which tryptase activates PAR-2	[123, 124]
		• Tryptase β has been reported to effectively detoxify various venoms	[125]
		• Since MCs are the only producers of tryptase and that tryptase is a component of MCETs, immunofluorescence microscopy to identify tryptase and DAPI staining together form the routine protocol to visualize MCETs.	[8]
	LL-37	• LL-37 is formed from an 18-kDa precursor protein (hCAP-18)	[126]
		• Other immune cells rather than MCs produce LL-37 including monocytes, neutrophils, MCs, NK cells, and B and T cells.	[126]
		• LL-37 possesses antimicrobial activity, induces the release of nucleic acids by MCs however, it has been reported not to play a role in formation of MCETs.	[61]
		• Its effectiveness against bacteria is due to its pore-forming activity	[62]

## Cell Death Pathways in Innate Immune

There are four cell death pathways described in innate immune cells when they are exposed to special bacteria and viruses including non-lytic and silent cell death mainly apoptosis, and inflammatory programmed lytic types including necroptosis, pyroptosis, and ETosis [31].

### Necroptosis

Engagement of TNF superfamily receptors, toll-like receptors (mainly TLR3 and TLR4), and interferon receptors drives the process of necroptosis during which the interaction between receptor-interacting protein kinase 1 (RIPK1) and RIPK3 leads in formation of heterodimer complex that promotes oligomerization of mixed-lineage kinase domain-like protein (MLKL)—acts as the RIPK3 substrate—through phosphorylation. MLKL oligomers translocate towards the plasma membrane and cause pore formation and further inflammatory response [32].

### Pyroptosis

The canonical pathway of pyroptosis is initiated when inflammasome sensor proteins mainly NLRP3 recognize the K^+^ efflux induced by microbial pathogens, toxins, and DAMPs [33]. Inflammasomes activated by DAMPs and PAMPs bind to apoptosis-associated speck-like protein (ASC) and recruit procaspase-1 and activate caspase-1. The latter molecule cleaves proIL-18 /1β and mediates the cleavage of gasdermin D (GSDMD). The N-terminal fragment of GSDMD (GSDMD-NT) mediates the formation of the pores in the plasma membrane, through which IL-18 /1β are released and water influx occurs. The final consequences of these molecular events are cell swelling and finally osmotic lysis [34].

### ETosis

**Fig. 3:**
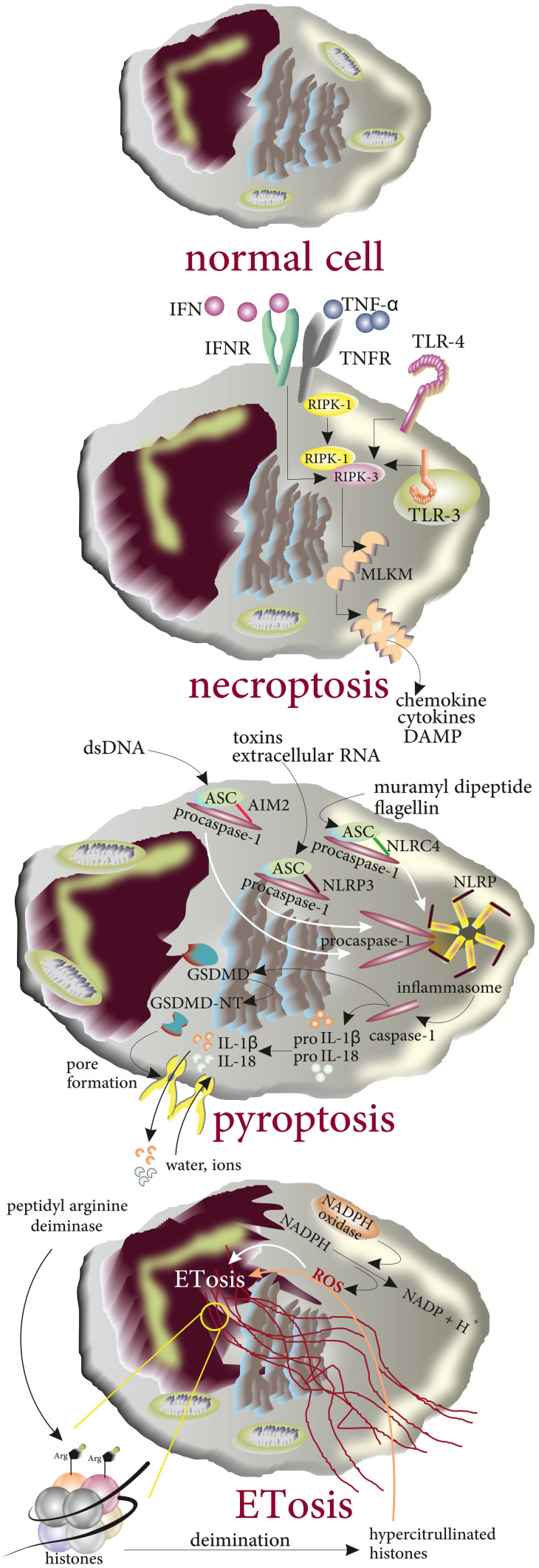
The molecular basis of inflammatory programmed lytic cell death types including necroptosis, pyroptosis, and ETosis. Engagement of TNFR, TLR3 and TLR4, and interferon receptors drives the interaction between receptor-interacting protein kinase 1 (RIPK1) and RIPK3 that promotes oligomerization of mixed-lineage kinase domain-like protein (MLKL) MLKL oligomers cause pore formation. In pyroptosis, inflammasomes activated by DAMPs and PAMPs bind to apoptosis-associated speck-like protein (ASC) and recruit procaspase-1 and activate caspase-1. Then caspase-1 cleaves proIL-18/1β and gasdermin D (GSDMD). The N-terminal fragment of GSDMD (GSDMD-NT) mediates the formation of the pores in the plasma membrane, through which IL-18/1β are released and water influx occurs. During ETosis, decondensation of chromatin, histone citrullination, and release of DNA into cytoplasm occur. DNA ejects into the extracellular space along with NET-associated antimicrobial peptides

In contrast to apoptosis, during ETosis, biologic changes such as nuclear condensation and DNA fragmentation do not happen. Indeed, nuclear chromatin decondensation in the cytoplasm is a common finding. Moreover, disintegration of the nucleus membrane, therefore cell death, results in release of nuclear DNA to form extracellular DNA nets [35]. From a molecular point of view, NADPH-oxidase-mediated production of ROS plays a key role in the formation of ETosis [36, 37]. Moreover, peptidyl arginine deiminase-mediated deimination of histone arginine residues to citrullines is another biochemical finding that contributes to chromatin decondensation [38]. Therefore, not interestingly hypercitrullinated histones are found in the structure of ETs when chemicals such as LPS and H_2_O_2_ act as the stimuli [39]. Since formation of ETs is followed by biologic changes including disintegration of the nuclear and cellular membranes, decondensation of chromatin and DNA structural modification mainly citrullination, and the release of both mitochondrial and nuclear DNA from the cells into the extracellular space, it is more likely that production of ETs results in the cell death [40] (Fig. 3).

## Extracellular Traps Formed by Immune Cells

### Neutrophils

**Fig. 4:**
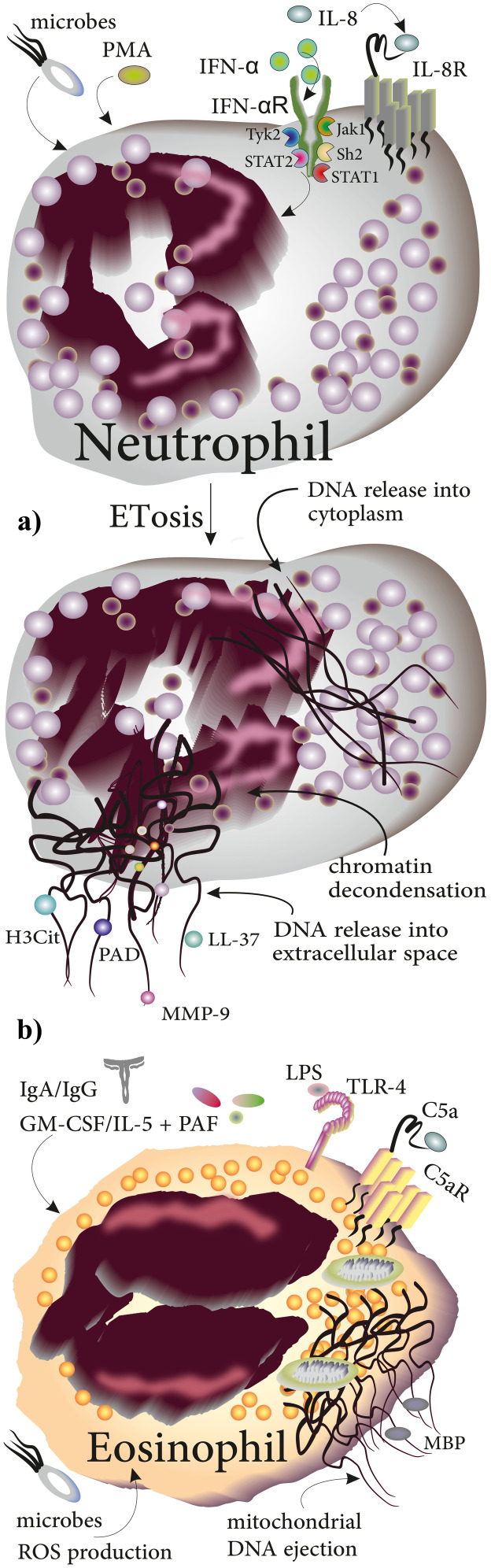
Production of ETs in neutrophils and eosinophils in response to chemical and biologic stimuli. **a** Stimuli including IL-8, IFN-α, PMA, and microbes induce the generation of NETs. Molecular events during ETosis include chromatin decondensation, DNA release into the cytoplasm, and release of DNA webs decorated with histones, LL-37, PAD, and MMP-9 into the extracellular space to trap invading pathogens. **b** Eosinophils produce EETs through releasing of mitochondrial DNA that becomes decorated with MBP upon exposure to stimuli including C5a, LPS, IgA/IgG, and GM-CSF/IL-5 + PAF

By studying inflammatory conditions including experimental shigellosis in rabbits and appendicitis in humans, Brinkmann and colleagues were the first to describe a novel extracellular anti-microbial mechanism in neutrophils in 2004. By staining histones, DNA, and neutrophil elastase, they reported the ability of neutrophils to eject DNA strands and utilize them for the trapping of pathogens [41]. A wide variety of stimuli including interferon (IFN)-α, interleukin (IL)-8, chemical agents (mainly phorbol myristate acetate; PMA), certain microbes, and their products have since been shown to induce the formation of neutrophil ETs (NETs) [42, 43]. NETosis is initiated by decondensation of chromatin, and the release of nuclear contents into the cytoplasm. In the final stage, DNA is released into the extracellular space to ensnare the invading pathogens [42]. Upon ejection of NETs, a variety of substances with bactericidal properties including proteases, LL-37, and protease-containing matrix metalloproteinase 9 (MMP-9) are released and contribute to the elimination of the pathogen [43]. Moreover, citrullinated histone H3 (H3Cit) and peptidyl arginine deiminase (PAD) are commonly released in conjunction with DNA [44] (Fig. 4a). NETosis is activated not only upon exposure to the above listed cytokines or chemicals but also the crosstalk of several cell types with neutrophils may induce the formation of NETs. Specifically, the production of NETs can be triggered by inorganic polyphosphate (polyP), notably also a secretory product of MCs which co-express it with CD68 [45]. The abundance of polyP expressing CD68^+^ MCs in the proximity of tumor cells in patients with colorectal cancer suggests that MCs may prime or trigger the production of NETs in cancer [45]. NETs have also been linked to procoagulant activity in patients with acute stroke. Indeed, the interaction between neutrophils and activated platelets induces the production and release of NETs decorated with phosphatidylserine (provides binding sites for the activation of coagulation factors when it is expressed on microvesicles or blood cells) [46]. Adhesion of coagulation factors and platelet-derived extracellular vesicles to NETs further contributes to the formation of thrombin and fibrin in stroke patients [46]. During NETosis, a variety of proteases are released from neutrophil granules and attach to DNA that have special biofunctions; for instance, neutrophil elastase, cathepsin G, and myeloperoxidase (MPO) are released from azurophilic (primary) granules, while lactoferrin and gelatinase are released from specific (secondary) granules and tertiary granules, respectively [41].

### Eosinophils

Over the recent years, it has become evident that neutrophils are not the only myeloid cells able to produce ETs. Release of eosinophil ETs (EETs) was first demonstrated when blood purified eosinophils were primed by IL-5 or IFN-γ for 20 min and then exposed to lipopolysaccharide (LPS) or complement factor C5a. In contrast to NETosis, EETosis results from the ejection of mitochondrial rather than nuclear DNA, presumably in a ROS–dependent manner [11]. Immobilized IgA/IgG and GM-CSF/IL-5 with platelet-activating factor (PAF) are among other stimuli of EETosis in vitro [47]. Additionally, formation of EETs may be triggered by the presence of viral infection as eosinophils derived from Ovalbumin-sensitized BALB/cJ mice were shown to produce EETs following infection with respiratory syncytial virus (RSV) in vitro [48]. The released EETs were composed of DNA decorated with toxic major basic protein (MBP) [49] (Fig. 4b). Production of EETs has been primarily studied in the context of severe eosinophilic asthma. Eosinophils from patients with severe asthma were reported to be more activated than those with non-severe asthma, and these eosinophils produce higher levels of ROS and EETs. Notably, the number of EET producing eosinophils correlates negatively with forced expiratory volume in 1 s (FEV1) and the severity of the disease [50], indicating the potential functional relevance of EETs in asthma. Production of EETs in asthmatics was, however, not affected by allergen challenge or levels of eotaxin, IFN-γ, and IL-5 in bronchoalveolar lavage [49]. Investigations of the structure of EETs showed that eosinophils release Charcot-Leyden crystals (CLCs) during the formation of EETs. CLCs are composed of eosinophil protein galectin-10 and commonly found in patients with allergic diseases such as asthma [51]. Non-stimulated eosinophils or those treated with diphenyleneiodium chloride were rarely found to release the crystals showing that crystals were associated with the formation of EETs. Considering the fact that formation of many crystals usually is associated with tissue injury, more studies are needed to clarify the significance of Charcot-Leyden crystals released during EETosis [47].

### Monocytes

**Fig. 5:**
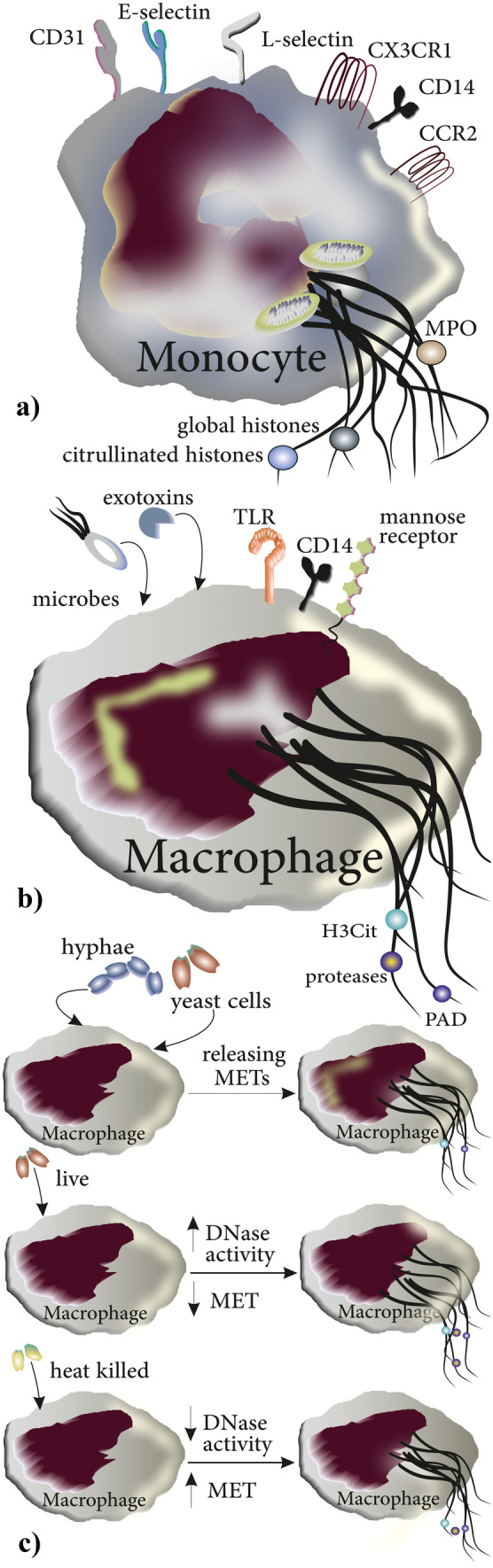
Production of ETs in monocytes and macrophages: **a** and **b** ETs produced by monocytes and macrophages are decorated with different types of peptides and proteins. **c** Both forms of *C. albicans*, yeast cells, and hypae induce the release of METs from macrophages. Additionally, both live and heat-killed *C. albicans* induce the formation of METs; however, heat-killed *C. albicans* are more potent in triggering MET formation from macrophages due to the absence of their DNase activity

Similar to eosinophils, monocytes can produce ETs by ejection of mitochondrial DNA that is decorated with global histones (H1, H2A/H2B, H3, H4) and citrullinated histones such as histone H4 citrullinated 3 (H4Cit3) [52]. Monocyte ETs (METs) can trap other cells, as demonstrated for spermatozoa from healthy individuals which showed a reduced mobility in the presence of monocytes simulated with *E. coli* [52]. Accordingly, monocytes have been found to be involved in microbial defense against parasites including viable *Besnoitia besnoiti* tachyzoites by the production of METs decorated with H3 histones and myeloperoxidase (MPO) [53]. In addition to humans, METosis has been reported in animals as well. In this regard, sensing of T. gondii-tachyzoites by monocytes of Harbour seals induces the formation of METs that results in entrapping and immobilizing of the parasite [54]. Notably, MET formation may also be induced by hormonal changes as demonstrated in a study of monocytes purified from peripheral blood of non-pregnant women during the menstrual cycle which showed that (a) more METs are produced during the luteal phase compared to the follicular phase and (b) revealed a positive correlation between the number of METs and serum levels of progesterone [55] (Fig. 5a).

### Macrophages

Both monocyte-derived macrophages and macrophage cell lines from humans and animals have been reported to produce and release macrophage extracellular traps (METs). Upon release, these web-like chromatin structures are decorated with H3Cit, granule proteases, and PAD similar to NETs [44]. Macrophages produce METs in response to a variety of microbes as well as to exotoxins of bacteria [56] (Fig. 5b) such as *Mannheimia haemolytica* (which causes bovine respiratory disease) and its leukotoxin or *E. coli-*derived hemolysin [56]. In macrophages stimulated with *M. haemolytica*, Aulik and colleagues showed that DNase treatment reduced the number of trapped and killed bacteria, thereby consolidating the role of METs in antimicrobial defense [56]. Consistently, Loureiro and coworkers demonstrated the role of METs in the control and killing of *C. albicans* in that (a) both forms of *C. albicans* yeast cells and hyphae can induce macrophages to produce METs and (b) both heat-killed and live *C. albicans* induce the generation of METs with the former more than the latter, possibly due to DNase activity in live *C. albicans* [57]. Importantly, this study introduces the DNase of *C. albicans* as a novel virulent factor. Indeed, METosis may act as a mechanism to confine the spread of *C. albicans* rather than killing the yeast [58] (Fig. 5c).

## Mast Cell Extracellular Traps

### Discovery and Early Reports

**Fig. 6:**
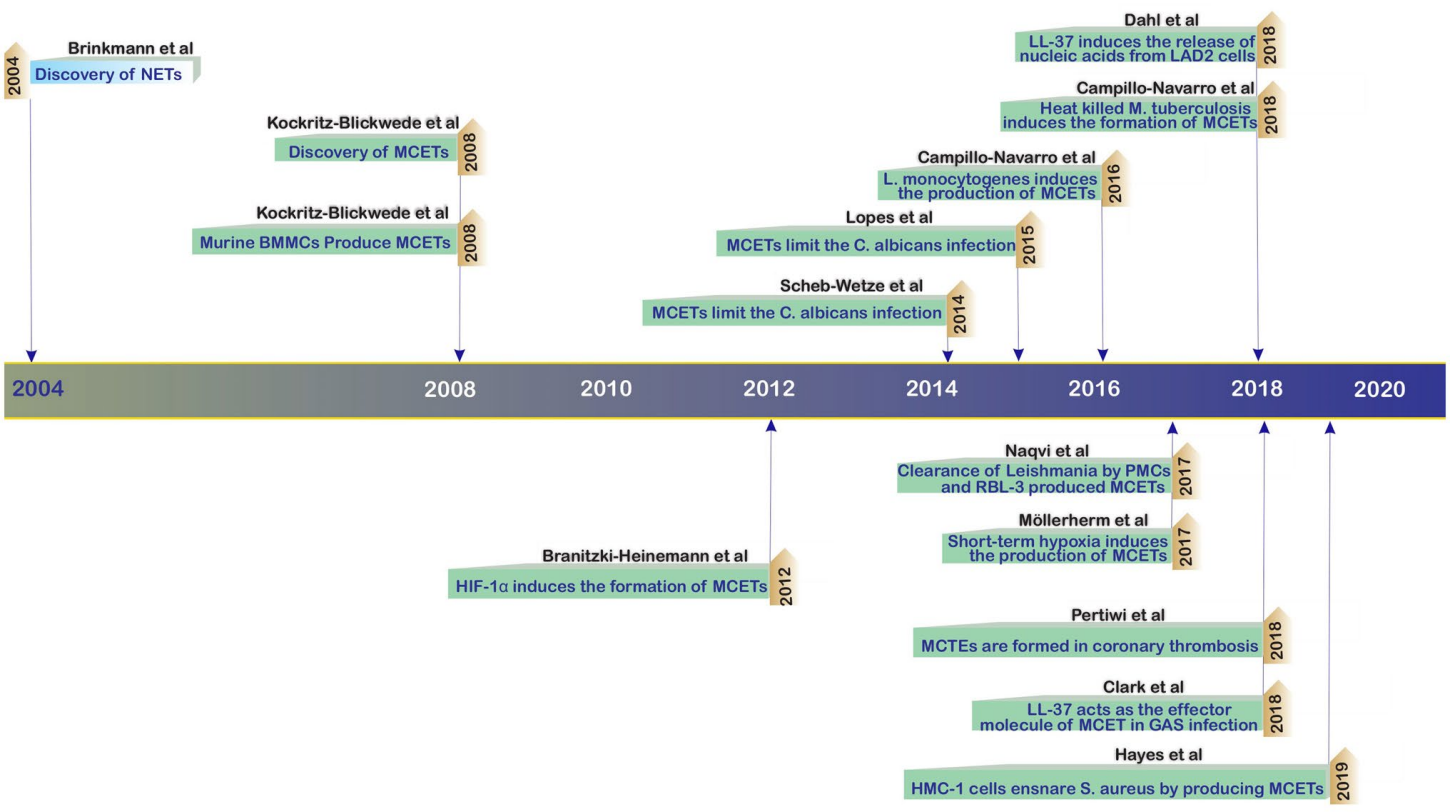
Timetable illustrating important discoveries in MCET biology. Four years after the first description of NETs by Brinkmann et al., MCETs were identified by Köckritz-Blickwede and colleagues in 2008. Over the past 5 years, a variety of stimuli for MCET formation have been described, including various microbes, microbial products, and chemicals, and several neoplastic MC cell lines and organ-derived MCs were reported to generate MCETs

Just 4 years after the discovery of NETs by Brinkmann et al. [41], Köckritz-Blickwede and colleagues reported that just like neutrophils, MCs can produce extracellular traps [12]. Over the past decade, the structure, function, and relevance of MCETs in infectious disease have been elucidated by models of intracellular or extracellular bacterial infection, fungi, or parasites. The timeline of these discoveries is highlighted in Fig. 6 [12, 30, 59–68].

### General Function and Composition of MCETs

The formation of MCETs is mainly considered to constitute an extracellular mechanism of host defense against invading pathogens [66]. A variety of stimuli have been found to induce the formation of MCETs, including several cytokines such as IL-23 and IL-1β that not only stimulate the formation of MCETs but also trigger MC degranulation [69]. Both intracellular and extracellular microbes, their products, can induce the release of MCETs [59]. Additionally, chemicals including PMA and endogenous molecules, namely, H_2_O_2_ and glucose oxidase induce the production of MCETs [12]. In contrast to eosinophils and monocytes which form small ETs from mitochondrial DNA, MCs similar to neutrophils form ETs via release of nuclear DNA [8, 11, 62, 70]. First studies provided insights into the structure of MCETs as web-like DNA strands decorated with histones, tryptase, and the cathelicidin LL-37 [12]. Cathelicidins serve as a group of peptides with antimicrobial properties and are produced by several immune cells, epithelial and genital cells [71]. LL-37 is a cationic peptide produced in humans from its precursor molecule hCAP18 by kallikreins [72]. LL-37 is produced by epithelial cells of various tissues [72, 73], and enhances the function of neutrophils, induces the production of inflammatory chemokines including IL-8, and induces tissue vascularization [74] by stimulating angiogenesis [75]. LL-37 can activate and degranulate LAD2 cells, a mast cell line, and hematopoietic CD34 + derived MCs expressing MrgX2, the receptor for LL-37 [76]. Immunohistochemical studies show that exogenous LL-37 is taken up by LAD2 cells and can be detected in their cytoplasm and nuclei. Treatment of LAD2 cells with LL-37 induces the release of nucleic acids and at high doses reduces the viability of the treated cells [61]. Following its release, LL-37 induces the expression of a variety of TLRs in MCs including both surface-expressed TLRs such as TLR2, TLR4, or TLR5, and endosomal TLRs including TLR7 and TLR9 [72]. In addition, LL-37 upregulates retinoic acid-inducible gene I (RIG-I)-like receptors (RLRs) and nucleotide-binding oligomerization domain (NOD)-like receptors (NLRs) in peritoneal MCs [77]. Another element of MCETs is tryptase which exerts its effects mainly by proteolytic cleavage of protease-activated receptor (PAR)-2 which is expressed in various immune cells but also in endo- or epithelial cells [78]. In addition to signaling via PAR-2, tryptase has additional important biofunctions as a protease including activation of matrix metalloproteinases and degradation of extracellular matrices [79].

### Imaging Techniques for the Study of MCETs

Prior to discussing the functional role of MCETs in microbial defense, we will briefly summarize the most relevant techniques used for the study of MCETs. Immunofluorescence and electron microscopy have been used successfully to demonstrate the ability of MCs to release ETs in the following protocol: (1) treatment of murine BMMCs (bone marrow–derived mast cells) or HMC-1 (Human Mast cell line-1) cells with PMA or glucose oxidase; (2) infection with *S pyogenes* that is either carboxyfluorescein labeled (if the imaging system is confocal microscopy) or unlabeled (if imaging is performed by electron microscopy); (3) fixation of cells using paraformaldehyde and washing in PBS for fluorescence microscopy; (4) applying antibodies against LL-37, tryptase, and histone; and (5) staining of DNA using DAPI (4,6-diamino-2-phenylindole) (or SYTOX-Green [59]) [12]. Alternatively, transmission electron microscopy has been used to study the structure and function of MCETs. To this end, samples are typically treated with MCET-inducing triggers such as PMA or bacteria and then fixed with glutaraldehyde-paraformaldehyde and osmium tetroxide. Following dehydration by ethanol samples are embedded in a mixture of ethanol-Epon and Epon resin. Additionally, uranyl acetate and lead citrate may be used to improve contrast [59]. Based on the finding that GreenGlo™ discriminates between nuclear DNA and strands of ETs by different excitation and emission wavelengths, Proust and colleagues developed a single-step protocol without washing to stain and discriminate these two types of DNA. Specifically in this protocol, nuclear DNA is detected by GreenGlo™ when excited at 470 nm with emission at 530 nm, while the same dye detects DNA strands of ETs at excitation of 350 nm and emission at 450 nm [80].

### Role in Microbial Defense

#### Role in Anti-bacterial Defense

**Fig. 7:**
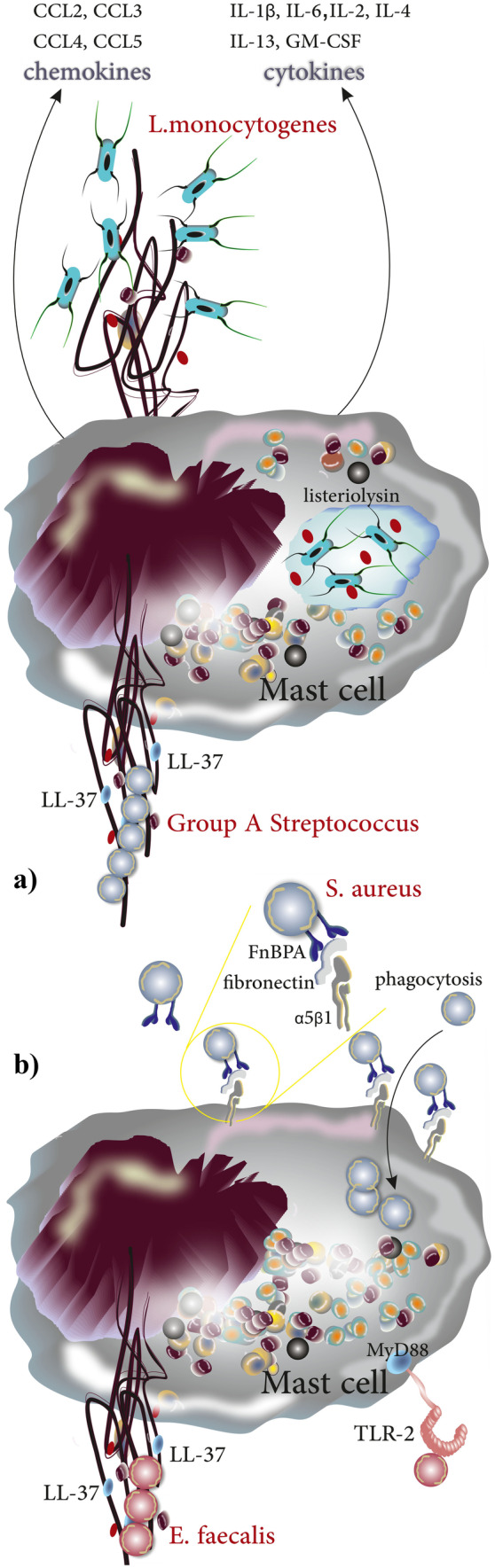
Production of MCETs in response to intra/extracellular bacteria. **a** After being phagocytosed, *L. monocytogenes* become trapped in the phagosome. Listeriolysin becomes activated at the acidic pH of the phagosome and lyses it, allowing *L. monocytogenes* to escape into the cytosol. MCs in return release a wide spectrum of cytokines and chemokines and produce MCETs. Group A Streptococcus (GAS) stimulates the production of MCETs with LL-37 playing a crucial role in the structure and function of the extracellular traps. **b** *S. aureus* induces the production of MCETs; however, it uses a molecular mechanism to evade elimination by MCETs in that it induces its phagocytosis into the MC cytoplasm through interaction of FnBPA/FnBPB on *S. aureus* with fibronectin (as bridging molecule) and α5Β1 on MCs. Additionally, TLR2 and MyD88 play a role in recognition and signaling, respectively, when MCs are exposed to *E. faecalis* and produce MCETs in response

In their first report of MCETs, Köckritz-Blickwede and colleagues observed that *S. pyogenes* become entrapped by extracellular structures around MCs [12]. Subsequent studies revealed that MCETs are formed in response not only to extracellular bacteria but also to intracellular bacteria including *L. monocytogenes*. MC activation upon exposure to *L. monocytogenes* is mediated largely by listeriolysin with MCs releasing in response a cocktail of cytokines, mainly IL-1β, IL-6, IL-2, IL-4, IL-13, GM-CSF, and a variety of chemokines including CCL2, CCL3, CCL4, and CCL5. Additionally, the release of osteopontin from activated MCs contributes to the clearance of the bacteria [81]. In parallel, *L. monocytogenes* induces the formation of MCETs in HMC-1 cells as demonstrated by the release of nuclear DNA and examination of the nuclear envelope showed the separation of the inner and outer membranes (Fig. 7a). *Enterococcus faecalis* infection has gained growing relevance due to resistance of the bacteria to various antibiotics and as the cause of nosocomial infection with a mortality rate of above 50% in critically ill hospitalized patients. Scheb-Wetzel and colleagues assessed the production and activation of MCETs in *E. faecalis* infected primary bone marrow–derived murine MCs using a β-hexosaminidase assay and toluidine staining. The authors also investigated the release of IL-6 and TNF-α (the release of which is dependent upon TLR-2) and showed that exposure to *E. faecalis* activates MCs and induces the degranulation and the release of IL-6 and TNF-α [66]. Injection of GFP-expressing *E. faecalis* into mice and subsequent immunohistochemical staining for CD117 identified an interaction of *E. faecalis* and MCs in vivo. Experimental *E. faecalis* infection in MCs derived from TLR2^−/−^ or MyD88^−/−^ mice further revealed the importance of TLR2 and MyD88 in the effective response to *E. faecalis* by the release of antimicrobial peptides. Moreover, application of endonuclease resulted in destruction of MCETs (and a partial growth inhibitory effect of MCs on *E. faecalis*) [66]. In parallel, the low rate of internalized *E. faecalis* by MCs indicated the involvement of an extracellular mechanism in the elimination of the pathogen. Immunostaining for histones next revealed that MCs cocultured with *E. faecalis* produce MCETs and confocal microscopy demonstrated that ensnared *E. faecalis* were killed, presumably as a direct consequence of their entrapment in MCETs [66]. MCETs also seem to play a role in the elimination of *S. aureus*, as both bone marrow–derived murine mast cells (BMMC) and HMC-1 have been shown to release MCETs upon exposure to this pathogen. Interestingly, the bacterium seems to induce its own phagocytosis (partially through interaction between MCs expressing α5Β1 and fibronectin-binding proteins FnBPA and FnBPB [82]) in an attempt to evade being trapped and killed in MCETs [83, 84] (Fig. 7b).

#### Role in Anti-fungal Defense

**Fig. 8:**
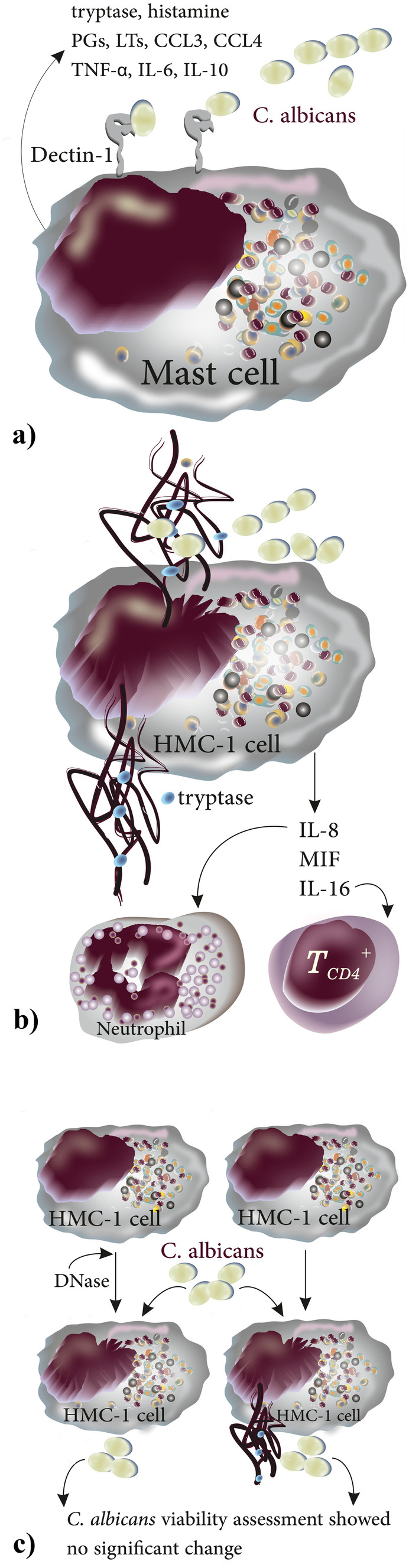
Main mechanisms used by MCs to control *C. albicans* infection. **a** *C. albicans* are recognized by MCs upon engaging MC surface expressed Dectin-1, MCs in turn release mediators including tryptase, histamine, PGs, LTs, CCL3, CCL4, TNF-α, IL-6, and IL-10. **b** Upon recognizing *C. albicans*, MCs become activated and degranulate and release IL-8 (neutrophil chemoattractant), MIF, and IL-16 (chemoattractant for CD4 + T lymphocytes); **c** comparing *C. albicans* viability either in the presence or absence of DNase showed no significant difference suggesting that MCET formation is not the main extracellular mechanism of *C. albicans* elimination

MCs can detect fungi like *C. albicans* via receptors such as β-glucan recognizing receptors (e.g., Dectin-1) and in response, release a variety of mediators including tryptase, histamine, prostaglandins (PGs), leukotrienes (LTs), and various cytokines, mainly CCL3, CCL4, TNF-α, IL-6, and IL-10 [65, 85] (Fig. 8a). The critical role of Dectin-1 in the recognition and response to *C. albicans* was highlighted in studies of cultured BMMCs from Dec^−/−^ mice, which showed only an impaired release of TNF-α, IL-6, and IL-13 as compared to control BMMCs following stimulation with *C. albicans* yeast and hyphae [86]. Lopes and colleagues studied the mechanisms by which MCs limit the growth of *C. albicans* and reported that MCs produce MCETs. By measuring β-hexosaminidase, the authors showed that MCs become activated and degranulate when exposed to *C. albicans*. *C. albicans*-infected HMC-1 cells were shown to release not only IL-8 (acting as neutrophil chemoattractant), macrophage migration inhibitory factor (MIF), and IL-16 (acting as chemoattractant for CD4 + T lymphocytes), but also MCETs evident as DNA decorated with tryptase after 7 h [65]. To investigate the impact of MCETs the authors applied DNase prior to infection (Fig. 8b). However, *C. albicans* viability did not differ significantly in the presence vs. absence of DNase, suggesting that although MCs could ensnare *C. albicans* by MCETs, this mechanism may not play a major role in fungal elimination [65] (Fig. 8c).

#### Role in Anti-parasitic Defense

Formation of MCETs has also been reported to play a role in the defense against parasites. To this end, Naqvi and coworkers investigated the elimination of *Leishmania donovani* and *Leishmania tropica* by peritoneal MCs (PMCs) and Rat Basophilic Leukaemia (RBL-2H3) cells. The authors reported a significant decrease in the viability rate of RBL-2H3 cells cocultured with either *L. tropica* or *L. donovani* promastigotes [63]. To probe for the release of MCETs, RBL-2H3 cells were seeded on cover slides and then co-cultured with carboxyfluorescein N-succinimidyl ester (CFSE) labeled promastigotes of *L. donovani* and *L. tropica* for 24 h. DNA was stained by DAPI, and fluorescently tagged antibodies were used to determine the presence of tryptase and histones. Treatment with DNase increased the viability of promastigotes demonstrating the functional relevance of MCETs in the anti-parasitic defense [63]. The results of this study showed that formation of MCETs was an extracellular mechanism used by MCs to eliminate leishmaniosis infection. However, coculturing RBL-2H3 with *L. donovani* and *L. tropica* could decrease the viability of the cells when compared to the control group after 18 h; in which, for example, coculturing the with *promastigotes of L. tropica* showed a decrease in cell viability (89.5% ± 2.5%; at 18 h and 79.3% ± 3.5% at 24 h) when compared to the control group (96.2% ± 3%). This group of researchers, to confirm the death of MCs during the production and the release of MCETs, investigated the presence of extracellular DNA using Sytox Green staining after co-culturing MCs with the promastigotes of *L. donovani* and *L. tropica.* Their results showed that only 2.3% ± 1.5% of MCs cultured in the absence of parasites released extracellular DNA after 18 h, while 6.5% ± 0.5% of the cocultured MCs with *L. tropica* did so. Interestingly, the rate increased and 21.6% ± 1.2% of MCs were reported to release extracellular DNA only after 24 h [63].

## Regulation of MCET Formation

As compared to the process of NETosis, the insight into the molecular mechanisms regulating the formation of MCETs is still sparse. In one of the few mechanistic studies, Möllerherm and colleagues recently demonstrated the formation of MCETs in response to short-term hypoxia (3 h). Notably, formation of MCETs in response to hypoxia was independent of hypoxia-inducible factor 1α (HIF-1α), a transcription factor that is critically involved in the adaptation to hypoxia. At normoxia, HIF-1α is rapidly degraded via the proteasome but stabilized when the cells experience hypoxia resulting in the transcription of hypoxia-regulated genes including erythropoietin, glucose transporters, glycolytic enzymes, antimicrobial factors, and VEGF [68]. While it has previously been reported that HIF-1α may induce the formation of MCETs [64], hypoxia caused MCET formation via a HIF-1α independent mechanism while suppressing the release of proinflammatory mediators including TNF-α, possibly in an attempt to attenuate the development of an inflammatory state and thus, to prevent tissue injury during hypoxia [64].

## Unmet Questions: Themes for Further Investigations

In this section, we highlight major unmet questions in the structure, biology, function, and regulation of MCETs as important topics for further investigations in the field (Table 2).

**Table 2:** Unmet questions: Themes for further investigations

Unmet questions in formation, structure, function, and regulation of MCETs	Ref
Formation of MCETs	
The molecular mechanism through which disruption of the nuclear membrane occurs in ETosis is still unknown. Notably, this mechanism may differ between neutrophils and mast cells, and as a function of the stimulus that triggers ETosis	[127]
The role of superantigens in the modulation of MCET formation deserves further investigation. It has previously been shown that Staphylococcal enterotoxin B (a superantigen expressed by *S. aureus*) induces the uptake of the bacterium. Considering that MCs produce MCETs to eliminate *S. aureus,* a better understanding of the effect of superantigens on MCETs formation and function may provide important insights into the mechanisms inducing or regulating MCET formation	[67]
The role of sterile inflammation in response to trauma, mechanical stress, or chemical challenge with respect to the induction of MCETs has so far not been addressed. Release of mitochondrial DNA in response to trauma can trigger the formation of NETs via a cyclic GMP‐AMP synthase and TLR-9 dependent pathway, suggesting a potential similar triggering role for MCETs that remains to be explored	[128]
Most recently, several papers suggested the activation of MCs during SARS-Cov-2 infection. Considering that MCs express ACE-2 (the critical receptor used by the virus to infect the host cells) and that MCs express receptors including endosomal TLRs to sense ds-RNA, they may play a role in the pathology of Covid-19. Although production of NETs in response to a variety of viruses has been reported, to the best of our knowledge, the production of MCETs in Covid-19 infection has not been investigated; therefore, it may be an interesting theme of research for other colleagues	[129–131]
Structure of MCETs	
The formation of MCETs and ejection of DNA decorated with proteins of which some act as autoantigens could potentially link MCETs to autoimmune diseases. Determining potential autoantigens released by MCETs may provide an interesting avenue for further investigations	[43]
While the exact role of histones in MCET is not yet clear, it has been shown that histones of NETs have cytotoxic effects like DAMPs. Conversely, extracellular histones induce the formation of NETs via interaction with TLR4/9 and application of anti-histone Abs like BWA3 could inhibit NET formation	[132]
The origin of DNA web of MCETs either nuclear or mitochondrial (or mixed) remains unanswered. A variety of specific markers could be used to define the origin of the DNA web of MCETs such as NADH-ubiquinone oxidoreductase chain 1 (Nd1) and cytochrome c oxidase subunit 1 (Cox1) as markers of mitochondrial DNA. Moreover, markers mainly glyceraldehyde 3-phosphate dehydrogenase gene (Gapdh) and actin beta (Actb) that are specific for nuclear DNA can be used to identify the nuclear DNA	[133]
Investigation of MC_TC_ formed MCETs in dermis of psoriasis plaques showed a colocalization of chymase and DNA suggesting that chymase may be a component of MCETs when they are produced by chymase positive MCs. Our knowledge regarding the biologic role of chymase in MCETs and maintaining its enzymatic activity upon binding to DNA web is poor, and more investigation is needed	[134]
Microbial evasion of MCETs	
The mechanisms by which pathogens aim to evade microbial defense by interrupting the formation and function of MCETs present an interesting topic for further investigations. For example, catalase deficiency supports the release of MCETs from MCs exposed to *Mycobacterium tuberculosis*, yet the role of catalase in other catalase-positive pathogens remains to be elucidated	[60]
Regulation of MCETs	
MCETs have been proposed to play an important role in coronary artery thrombosis; however, this potentially important pathogenic aspect remains to be resolved	[8]
NETs have previously been implicated in the pathogenesis of autoimmune diseases including systemic lupus erythematosus (SLE) as NETs are decorated by matrix metalloproteinase-9 (MMP-9) which upon release activates endothelial MMP-2 and induces endothelial damage in SLE. MCs likewise produce several MMPs including MMP-9, yet their possible involvement in autoimmune diseases including SLE remains to be addressed	[110]
A pathogenic role for MCs in psoriasis via formation of MCETs and release of IL-17 upon stimulation with IL-23 and IL-1β has been proposed. The role of MCETs in other pathologies dominated by MC-released cytokines like IL-17 awaits further study	[69]
To the best of our knowledge, no investigation has so far addressed the formation of MCETs in individuals with cutaneous or systemic mastocytosis. A potential propensity or inability of neoplastic MCs to form MCETs in response to trauma, sterile inflammation, or microbes may reveal new mechanistic insights that may underlie or contribute to the pathological features of the disease	[57]
The pattern of NETosis regulation upon engagement of innate immune receptors has been previously investigated. Engagement of Dectin-1 (a receptor involved in the recognition of chitin as a biopolymer in the structure of fungi) upon exposure to *Candida albicans* but not to that efficacy to hyphae drives phagocytosis to elimination of the pathogen suggesting that Dectin-1 suppresses the NETosis and contributes to orchestration of innate immune response according to the size of the pathogen; the result of this experiment was supported when Dectin^−/−^ neutrophils showed an aberrant production of NETs. MCs express Dectin-1, but its regulatory role on the production of MCETs needs to be investigated	[135–137]

## Summary and Conclusion

Following the initial discovery of NETs in 2004, a similar ability for ETosis—albeit at a smaller scale—was demonstrated in various myeloid cells including eosinophils and monocytes by ejection of mitochondrial DNA. In contrast, mast cells seem to be the only other immune cell identified so far that is—similar to neutrophils—able to form ETs from nuclear DNA. Engagement of receptors by various ligands and also chemicals induces the formation of MCETs. The main inducers and involved receptors are listed in Table 3.

**Table 3:** The main receptors and chemicals that are capable of inducing the formation of MCETs

Chemical inducers of MCET formation	Specification, mechanism, and involved diseases	Ref
phorbol-12-myristate-13-acetate (PMA)	• Primarily was isolated from unripe fruit of *Sapium indicum* (a mangrove plant from Euphorbiaceae family). PMA is a highly pro-inflammatory agent and tumor promoter.	[138]
	• As a general protocol, treatment of MCs with PMA before infection stimulates the production of MCETs.	[12]
Glucose oxidase	• Catalyzes the production of H_2_O_2_	[12]
Cytokines as inducers of MCET formation		Ref
IL-23	• induces MC degranulation and production of MCET in human skin and induces the release of IL-17 which is involved in psoriasis	[134]
IL-1β	• induces MC degranulation and production of MCET in human skin and induces the release of IL-17 which is involved in psoriasis	[134]
Receptors involved in MCET formation		Ref
Dectin-1?	• MCs recognize the presence of fungi including candida mainly using Dectin-1 dependent pathway and this receptor has been previously shown to have a role in NETosis and production. It is likely that Dectin-1 may have a similar role in production of MCETs	[27, 139]
TLR-2?	• MCETs formation is dependent on NADPH oxidase mediated production of ROS, and TLR-2 signaling plays a role in production of ROS. It is now clear that neutrophils recognize several pathogens using TLR-2 and produce NETs in turn; since MCs express TLR-2, the receptor is likely involved in production of MCETs, but it has not been specifically investigated.	[140, 141]

It should be noticed that the shape of MCETs seems to differ according to the local tissue and testing environment which should be considered in the interpretation of results. Specifically, MCETs in skin specimen are more compact when compared to those formed in vitro [69]. Protocols for the investigation of ETs are overall similar for different innate immune cell populations, and the function of ETs is largely determined by the bioactivity and biofunction of peptides decorating the ejected DNA strands. While our understanding of their physiological and pathogenic role is still rudimentary—as compared to the well-established role of NETs—MCETs have recently become implicated in host defense as well as various autoimmune, cardiovascular, or pulmonary disorders. Like NETs, MCETs act as scaffolds composed of nuclear DNA and peptides with antimicrobial activity that act as extracellular mechanism for trapping and killing of invading pathogens. Although the production of extracellular traps by immune cells has been predominantly linked to antimicrobial defense, some lines of evidence suggest a link to other pathologic conditions. For instance, MCs have been found to infiltrate and degranulate in skeletal muscles in autopsy samples of patients with amyotrophic lateral sclerosis (ALS) and are associated with NET producing neutrophils by recruiting them via the release of chymase that acts as neutrophil chemoattractant. Interestingly, the application of masitinib (a widely used tyrosine kinase inhibitor) could suppress the axonal pathology and secondary demyelination in ALS by suppressing MCs and interference with their role in neutrophils recruitment [87]. Analogously, MCs have been shown to infiltrate lung and vascular tissue in pulmonary hypertension and lung fibrosis [88]. Notably, pathological remodeling in these diseases could be attenuated or prevented not only by mast cell stabilizers or in mast cell deficient animals [89, 90], but also—at least in vitro—by DNase treatment [91, 92], suggesting a potential pathogenic contribution of MCETs. Interrupting the formation of MCETs may also act as a successful strategy of pathogens to evade the MC-mediated immune response. Along these lines, MCET formation can be detected following stimulation with heat-killed *Mycobacterium tuberculosis*, yet not in response to its viable counterparts, as catalase from *Mycobacterium tuberculosis* seems to prevent MCET formation by degrading hydrogen peroxide [60]. Other pathogens such as *C. albicans* may evade entrapment by MCETs by expressing DNase as a virulence factor [57]. At present, our understanding of MCETs, their formation, and structure, as well as their involvement in microbial defense and non-infectious pathologies, is only beginning to emerge. Although formation of ETs including MCETs is likely to be a late response but effective one against the presence of intruding microorganisms, however, the release of DNA into extracellular space may orchestrate the immune responses such as the production of anti-citrullinated protein antibodies in seropositive rheumatoid arthritis. Not surprisingly, since many AMPs attached to DNA web should be normally restricted in cytoplasmic granules, their release may have harmful effects such as degrading ECM or activating tissue-destructive mechanisms [93, 94]. Better insight into the function and regulation of MCETs, as well as the mechanisms by which pathogens tend to evade MCET-mediated elimination may provide not only important biological insights but pave the way for novel interventions in infectious, autoimmune, and other mast cell-related diseases.

## Abbreviations

BMMCs: Bone marrow derived mast cells
DAPI: 4,6-Diamino-2-phenylindole
ET: Extracellular trap
GAS: Group A Streptococcus
H3Cit: Citrullinated histone H3
HIF-1α: Hypoxia-inducible factor 1α
HMC-1: Human Mast cell line-1
HMDM: Human monocyte–derived macrophage
mAb: Monoclonal antibody
MCETs: MCs extracellular trap
mDCs: Myeloid dendritic cells
MPO: Myeloperoxidase
NOD: Nucleotide-binding oligomerization domain
PAD: Peptidyl arginase deiminase
PAD: Peptidyl arginine deiminase
PMA: Phorbol-12-myristate-13-acetate
RIG-I: Retinoic acid-inducible gene I
ROS: Reactive oxygen species
RSV: Respiratory syncytial virus
NLRP3: NOD-like receptor protein 3
ASC: Apoptosis-associated speck-like protein
